# Assessment of Cd(II) adsorption capability and mechanism from aqueous phase using virgin and calcined lignin

**DOI:** 10.1016/j.heliyon.2020.e04298

**Published:** 2020-06-25

**Authors:** Fumihiko Ogata, Eri Nagahashi, Hirona Miki, Chalermpong Saenjum, Takehiro Nakamura, Naohito Kawasaki

**Affiliations:** a Faculty of Pharmacy, Kindai University, 3-4-1 Kowakae, Higashi-Osaka, Osaka, 577-8502, Japan; b Faculty of Pharmacy, Chiang Mai University, Suthep Road, Muang District, Chiang Mai, 50200, Thailand; c Cluster of Excellence on Biodiversity-based Economics and Society (B.BES-CMU), Chiang Mai University, Suthep Road, Muang District, Chiang Mai, 50200, Thailand; d Antiaging Center, Kindai University, 3-4-1 Kowakae, Higashi-Osaka, Osaka, 577-8502, Japan

**Keywords:** Adsorption, Cadmium ion, Calcination, Lignin, Bioengineering, Surface chemistry, Environmental chemistry, Environmental engineering, Environmental pollution, Chemistry, Environmental science

## Abstract

Herein, to assess the adsorption capability and elucidate the adsorption mechanism of Cd(II) from the aqueous phase, virgin lignin (Lig) and calcined lignin (Lig200, Lig400, Lig600, Lig800, and Lig1000) were prepared. The characteristics, including specific surface area and pore volume of adsorbents, were investigated, and the adsorption capability along with the effect of temperature, contact time, and pH on the adsorption of Cd(II) were evaluated. The characteristics of the adsorbent surface were related to the adsorption capability of Cd(II) from the aqueous phase, and the correlation coefficients between the adsorbed amount and specific surface area and total pore volumes were 0.872 and 0.960, respectively. Moreover, the amount adsorbed using Lig800 (91.3 mg/g) was higher than that using other adsorbent samples. The adsorption mechanism was elucidated to investigate the binding energy and elemental distribution before and after Cd(II) adsorption. Finally, the desorption capability of Cd(II) from Lig800 using a hydrochloric acid solution was demonstrated. Results obtained herein suggest that Lig800 is a potential candidate for the removal of Cd(II).

## 1 Introduction

Since 2015, 17 sustainable development goals (SDGs, the 2030 Agenda for Sustainable Development) have been announced. Among them, several SDGs are strongly and directly related to “water environments.” Therefore, water pollution such as eutrophication, contamination by heavy metals, and inorganic nitrogen are serious problems and are a growing concern worldwide. In particular, cadmium is an important water environmental pollutant derived from industries (human activity) such as plating and mining (Alloway, 1995; Kabata-Pendias and Pendias, 2001). Additionally, cadmium (Cd(II)) and cadmium compounds are listed in Group 1 (carcinogenic to humans) by the International Agency for Research on Cancer. As free Cd(II) is highly toxic to the ecosystem, humans, and animals (Ursinyova et al., 2000), the maximum contamination level of Cd(II) is defined as 0.005 mg/L by the United States Environmental Protection Agency. Therefore, the removal of Cd(II) from water environments plays a vital role in purifying wastewater and preventing human illness.

The natural abundance and characteristic ability of biosorbents derived from waste biomass to interact with metal ions in aqueous solution systems have been reported by previous literatures (Chen and Wu, 2004; Gérente et al., 2000). Previous studies have also reported that the interaction between biosorbents and metal ions is related to complexation, coordination, ion exchange, and adsorption (Ge and Li, 2018; Supanchaiyamat et al., 2019; Volesky, 1990). Among biosorbents, lignin as an adsorbent has garnered greater interest worldwide. Lignin, an insoluble three-dimensional polymer containing oxygen functional groups, is the second most abundant natural organic substance together with cellulose and hemicellulose (Ge and Li, 2018; Guo et al., 2008). Oxygen-containing functional groups include carboxyl, hydroxyl, and benzyl alcohol groups. In addition, previous studies reported the complex formation between Cd(II) and functional groups such as carboxyl or phenolic groups of raw lignin (Dzomback and Morel, 1990; Guo et al., 2008). Therefore, oxygen-containing functional groups are crucial factors for the removal of Cd(II) from the aqueous phase. Additionally, many researchers have focused on the physicochemical properties of raw lignin derived from waste biomass or plant biomass. As an adsorbent, it has received considerable attention for the removal of heavy metal ions (inorganic compounds) or organic compounds from aqueous solutions (Guo et al., 2008; Sarkanen and Ludwig, 1971). Moreover, the physical and chemical properties of modified lignin have been investigated, and its adsorption capability for noble or heavy metal ions, organic compounds (dyes), and gases has been evaluated by many researchers (Celik and Demirbas, 2005; Dizhbite et al., 2013; Huang et al., 2019; Parajuli et al., 2005; Peternele et al., 1999; Supanchaiyamat et al., 2019; Wang et al., 2020). Among the modification methods of lignin, calcination is one of the most useful to improve the adsorption potential for waste biomass (Ogata et al., 2018, 2020).

Berrima et al. (2016) reported the adsorption capability of heavy metals using charcoal from lignin (the calcination temperature was only 600 °C). Calcination treatment directly affects the physicochemical properties, indicating that the adsorption capability could be significantly changed by the calcination conditions. In particular, the calcination of lignin causes the elimination of heteroatoms such as oxygen and hydrogen; then, the different forms of layered aromatic planar structure are produced. These structures are arranged in an irregular manner, leaving interstices due to the calcination conditions (Berrima et al., 2016). Therefore, the evaluation of properties of calcined lignin at different temperatures and assessment of the adsorption capability of heavy metals was crucial.

However, there is a lack of reports on the adsorption capability of Cd(II) from the aqueous phase using calcined lignin prepared at different temperatures. Therefore, if the adsorption potential of calcined lignin, which is waste biomass, at different temperatures is explored, its value and applicability would be increased.

This study aims to investigate the characteristics of calcined lignin at different temperatures, focusing on its adsorption capability, temperature effect, contact time, and pH in the adsorption of Cd(II) from an aqueous solution using calcined lignin. In addition, the adsorption mechanism of Cd(II) is also investigated.

## 2 Materials and methods

### 2.1 Materials

Lignin (Lig) was purchased from Tokyo Chemical Industry Co., Ltd. (Tokyo, Japan). Calcined Lig at 200, 400, 600, 800, and 1000 °C was prepared by keeping it in a muffle furnace for 2 h (denoted as Lig200, Lig400, Lig600, Lig800, and Lig1000, respectively). Cadmium chloride was purchased from FUJIFILM Wako Pure Chemical Co. (Osaka, Japan).

The morphologies of each adsorbent were measured by scanning electron microscopy SU1510 (SEM, Hitachi High-Technologies Co., Tokyo, Japan). The specific surface area and pore volumes were analyzed using a specific surface analyzer NOVA4200*e* (Quantachrome Instruments Japan G.K., Kanagawa, Japan). The surface functional groups were analyzed by the Fourier-transform infrared (FT-IR) spectroscopy system 460Plus (JASCO Co., Tokyo, Japan). The binding energy and elemental distribution of the adsorbent surface were measured by the X-ray photoelectron spectroscopy system AXIS-NOVA (Shimadzu Co., Ltd., Kyoto, Japan) and electron microanalyzer JXA-8530F (JEOL, Tokyo, Japan), respectively.

### 2.2 Removal of Cd(II) using virgin and calcined Lig prepared at different temperatures

Adsorbents (0.05 g), namely Lig, Lig200, Lig400, Lig600, Lig800, and Lig1000, were mixed with a Cd(II) solution of 50 mL at 100 mg/L. The reaction mixtures were shaken at 100 rpm for 24 h at 25 °C and then filtrated by a 0.45 μm membrane filter. Inductively coupled plasma-optical emission spectrometry (ICP-OES) (iCAP 7600 Duo, Thermo Fisher Scientific Inc., Kanagawa, Japan) was used to measure the concentration of Cd(II) in the obtained filtrate. The quantity of Cd(II) adsorption was calculated using the levels before and after each experiment.

### 2.3 Effect of temperature, contact time, and pH on the adsorption of Cd(II) using Lig and Lig800

First, to confirm the effect of temperature, 0.05 g of adsorbents samples, namely Lig and Lig800, were mixed with a Cd(II) solution of 50 mL at 5, 10, 20, 30, 40, 50, 80, 100, and 300 mg/L. The reaction mixtures were shaken at 100 rpm for 24 h at 25 and 50 °C. Second, to confirm the effect of contact time, the same adsorbent samples (0.05 g) were mixed with a Cd(II) solution of 50 mL at 100 mg/L. The reaction mixtures were then shaken at 100 rpm for 1 and 10 min as well as for 0.5, 1, 2, 6, 22, and 24 h at 25 °C. Finally, to confirm the effect of pH, the same adsorbent samples (0.05 g) were mixed with a Cd(II) solution of 50 mL at 100 mg/L. The solution pH was adjusted from 3 to 6 by adding either hydrochloric acid or sodium hydroxide solution (FUJIFILM Wako Pure Chemical Co., Osaka, Japan). The reaction mixtures were shaken at 100 rpm for 24 h at 25 °C. The adsorption capacity on Cd(II) was calculated using the levels before and after each experiment. The student's *t*-test was used for a comparative analysis of two groups. A minimum *p*-value of 0.05 (*p* < 0.05) was chosen as the significance level.

### 2.4 Desorption capability of Cd(II) from Lig800 using hydrochloric acid solution

First, 0.5 g of Lig800 was mixed with 300 mg/L of a Cd(II) solution in the volume of 500 mL. The reaction mixtures were shaken at 100 rpm at 25 °C for 24 h and then filtrated through a 0.45 μm membrane filter. The level of Cd(II) in the obtained filtrate was measured by ICP-OES. The adsorption quantity on Cd(II) was calculated using the levels before and after each experiment. After adsorption, Lig800 was collected, dried, and used for the further desorption experiment. The collected Lig800 (0.5 g) was then mixed with a hydrochloric acid solution of 50 mL at 1 and 10 mmol/L. The reaction mixtures were shaken at 100 rpm for 24 h at 25 °C and then filtrated through a 0.45 μm membrane filter. The quantity of Cd(II) desorbed from Lig800 was also calculated using the level before and after the desorption experiment. The results of this study are expressed as means ± standard errors (*n* = 2–3, Sections 2.2–2.4).

## 3 Results and discussion

### 3.1 Effect of calcination on physical properties

**Figure 1:**
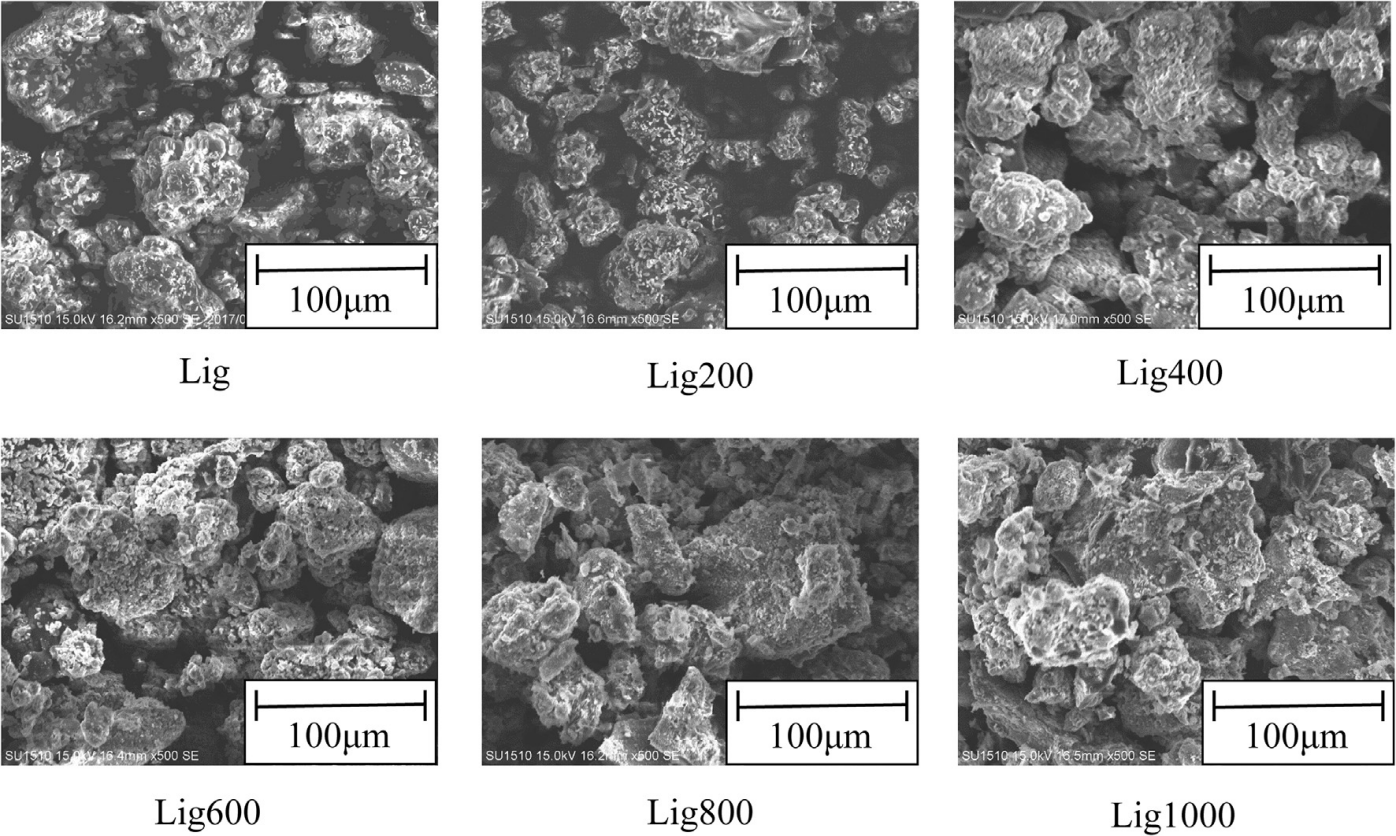
SEM images of adsorbents.

Figure 1 shows SEM images of the adsorbents. The roughness on the adsorbent surface was observed in each sample. A previous study reported that lignin had a roughness that was not aggregatory (Ogunsile and Bamgboye, 2017); similar trends were observed in the present study. Additionally, several small pores were observed in Lig600, Lig800, and Lig1000 under our experimental conditions. (The relationship between the amount of Cd(II) adsorbed and properties of Lig samples is discussed in Section 3.2).

Table 1 illustrates the physical properties of the adsorbents. The yield percentage decreased with the increase of the calcination temperatures. Conversely, the specific surface area, micropore volume (diameter lower than 2 nm), mesopore volume (diameter between 2 and 50 nm), and total pore volume increased with the increase of the calcination temperatures. These phenomena suggest the occurrence of carbonization and removal of the volatile substances and metallic impurities. The values of Lig800 were higher than those of the other adsorbents. Interestingly, those of Lig1000 were lower than those of Lig800, indicating that Lig1000 was degraded by carbonization at approximately 1,000 °C. A similar phenomenon was observed using waste wood (organic material) (Yokoyama et al., 2008). Pore size distribution is important for the removal ability of adsorbate from the aqueous phase (Oishi et al., 2011). The current study revealed that Lig800 might be the most potent for the removal of Cd(II) from the aqueous phase (Shimakami et al., 2005).

**Table 1:** Physical properties of adsorbents.

Adsorbents	Yield (%)	Specific surface area (m^2^/g)	Pore volume (μL/g)			
			*d*≦2 nm	2 < *d*≦50 nm	*d* > 50 nm	Total
Lig	100	-	0.0	0.8	1.0	1.8
Lig200	85	0.2	0.0	0.2	0.0	0.2
Lig400	57	11.7	0.2	2.6	0.0	2.8
Lig600	47	176.5	1.1	9.1	0.0	10.2
Lig800	43	306.3	1.4	21.1	1.4	23.9
Lig1000	36	82.7	0.3	10.8	0.0	11.1

**Figure 2:**
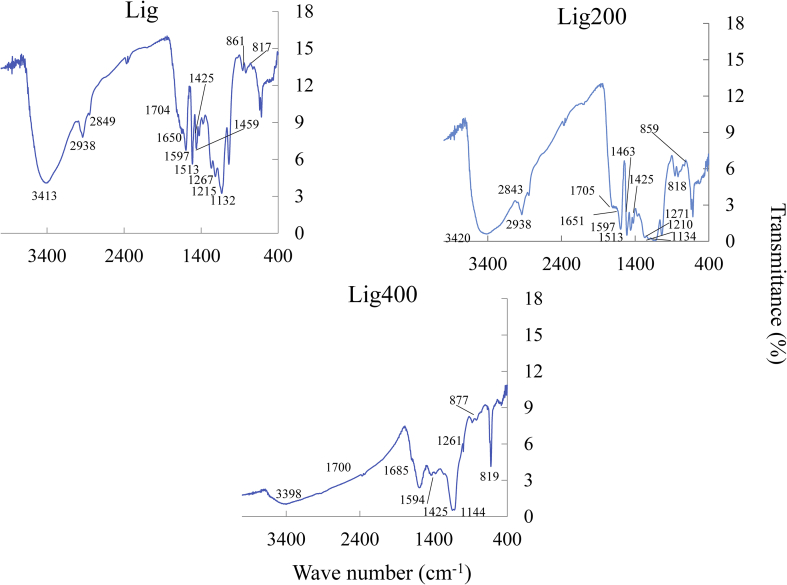
FT-IR spectroscopy images of Lig, Lig200, and Lig400.

Additionally, Figure 2 shows the FT-IR spectrum of Lig, Lig200, and Lig400. The bands at 3413 (H–O–H stretching in phenolic or aliphatic groups), 2938, and 2849 (C–H stretching in methyl and methylene groups), 1704 (C=O–OH stretching in unconjugated carbonyl groups), 1650 (C=O stretching in conjugated carbonyl groups), 1597, 1513, 1425 (aromatic ring bending), 1267, 1215, 1132, 861, and 817 cm^−1^ (syringyl ring stretching or bending) in Lig were detected. Lig has an aromatic three-dimensional polymer structure, including functional groups such as phenolic, benzyl alcohol, hydroxyl, carboxyl, methoxyl, and aldehyde groups. Moreover, these intensities showed that phenolic units were more abundant than carboxyl groups in Lig (Guo et al., 2008; Nagahashi et al., 2018; Sarkanen and Ludwig, 1971; Tejado et al., 2007). The intensities of the bands in Lig200 and Lig400 decreased with the increase of the calcination temperatures. These results indicate that the surface functional groups of Lig200 and Lig400 decreased. In contrast, the signals of Lig600, Lig800, and Lig1000 were not detected because Lig600, Lig800, and Lig1000 were prepared as carbonaceous material from lignin by the calcination treatment under our experimental conditions. Previous studies have reported that the number of surface functional groups such as phenolic and carboxyl groups decreased upon carbonization at approximately 600 °C (Yokoyama et al., 2008; Uematsu et al., 2020). Therefore, similar trends were observed herein.

### 3.2 Removal of Cd(II) by virgin Lig and calcined Lig at different temperatures

The quantities of Cd(II) adsorbed onto Lig, Lig200, Lig400, Lig600, Lig800, and Lig1000 were 26.3, 30.1, 26.9, 46.4, 91.3, and 69.1 mg/g, respectively. The adsorption capabilities were in the order of Lig≒Lig200≒Lig400 < Lig600 < Lig1000 < Lig800, indicating that Lig800 is the most suitable adsorbent for the Cd(II) removal from the aqueous phase under our experimental conditions. Table 2 shows the comparison of Cd(II) adsorption capacity of Lig or Lig800 with the other reported adsorbents (Berrima et al., 2016; Borah and Sennapati, 2006; Dizhbite et al., 2013; Harmita et al., 2009; Naiya et al., 2009; Njikam and Schiewer, 2012; Parajuli et al., 2005; Kumar et al., 2009). The amount of Cd(II) adsorbed using Lig800 was greater than that of the other adsorbents (except for crosslinked lignocatechol and pyrite). These results indicate that Lig800 was a high potential candidate for the Cd(II) removal from the aqueous phase. Additionally, the Cd(II) adsorption capability of virgin or modified lignin was lower than that using Lig800. It can be suggested here that calcination is one of the useful treatments to enhance the Cd(II) adsorption capability of using lignin.

**Table 2:** Comparison of Cd(II) adsorption capacity of Lig or Lig800 with other reported adsorbents.

Samples	Adsorption capability (mg/g)	pH	Temperature (°C)	Initial concentration (mg/L)	Contact time (h)	Adsorbent (g/L)	References
Activate alumina	35.06	5	30	50	2	7.5	Kumar et al., 2009
Crosslinked lignocatechol	Approximately 130	5.2	30	22.4	24	1.3	Parajuli et al. (2005)
Charcoal from lignin	72.9	5	35	-	3	1	Berrima et al. (2016)
Citrus peels	Approximately 80	5	-	100	3	1	Njikam and chiewer, 2012
Pyrite	166.0	6	30	100	1	-	Borah and Senapati (2006)
Modified lignin	35.9	5	20	112	24	10	Dizhbite et al. (2012)
Original lignin	2.06	6.5	24	5	7	3.3	Harmita et al. (2009)
Lig	26.3	5.3	25	100	24	1	This study
Lig800	91.3	5.3	25	100	24	1	This study

**Figure 3:**
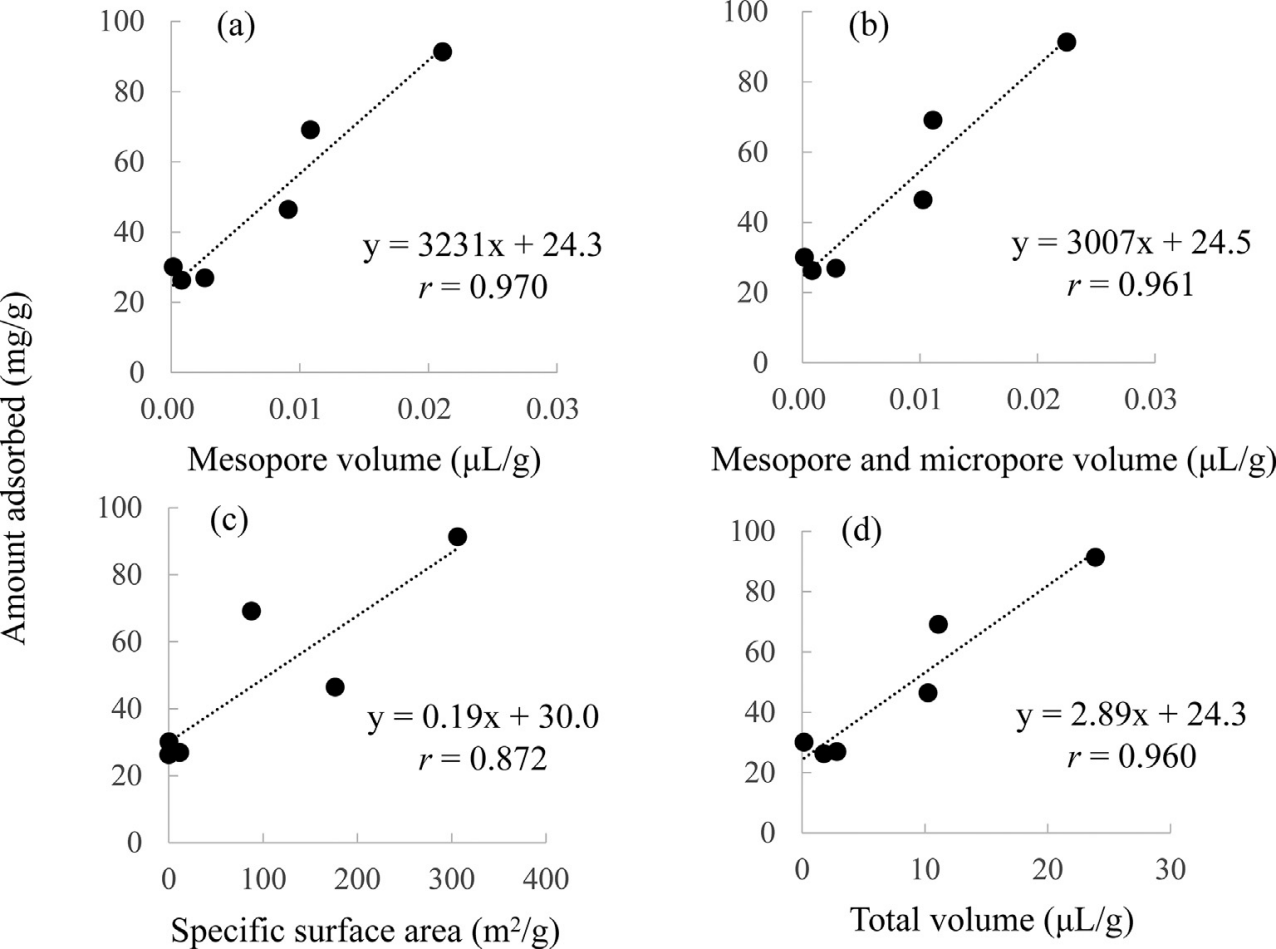
Relationship between the amount of Cd(II) absorbed and physical properties of adsorbents. (a) y = 3231x + 24.3, *r* = 0.970, (b) y = 3007x + 24.5, *r* = 0.961, (c) y = 0.19x + 30.0, *r* = 0.872, (d) y = 2.89x + 24.3, *r* = 0.960.

Furthermore, the relationship between the amount adsorbed and the characteristics of adsorbents was evaluated (Figure 3). The correlation coefficients between the amount adsorbed and mesopore volume, mesopore volume plus micropore volume, total volume, and specific surface area were 0.970, 0.961, 0.960, and 0.872, respectively. These results indicate that the adsorption of Cd(II) using lignin was strongly related to the adsorbent (lignin) surface properties. The micropores and mesopores are the major contributors to the adsorption capability for adsorbates from the aqueous phase. Thus, these properties control the interaction between adsorbate and adsorbent. Herein, micropore, mesopore, and specific surface area of Lig600, Lig800, and Lig1000 were higher than those of other Ligs. Calcined lignin at over 600 °C should show a high adsorption capability due to the merits such as high specific area, well-developed pore channels, and volumes (Hartmann et al., 2005; Suzuki, 1990; Zhou et al., 2016). Previous researches have reported that surface functional groups such as carboxyl or hydroxyl groups and surface charge of adsorbent strongly affect the adsorption capability of Cd(II) using lignin from the aqueous phase (Berrima et al., 2016; Ge and Li, 2018; Supanchaiyamat et al., 2019). As these factors were affected by the pH of the solution, our study evaluated the effect of solution pH on the adsorption of Cd(II) in the following Section 3.5. In the following experiments, Lig and Lig800 were selected to elucidate the adsorption mechanism of Cd(II).

### 3.3 Adsorption isotherms of Cd(II)

**Figure 4:**
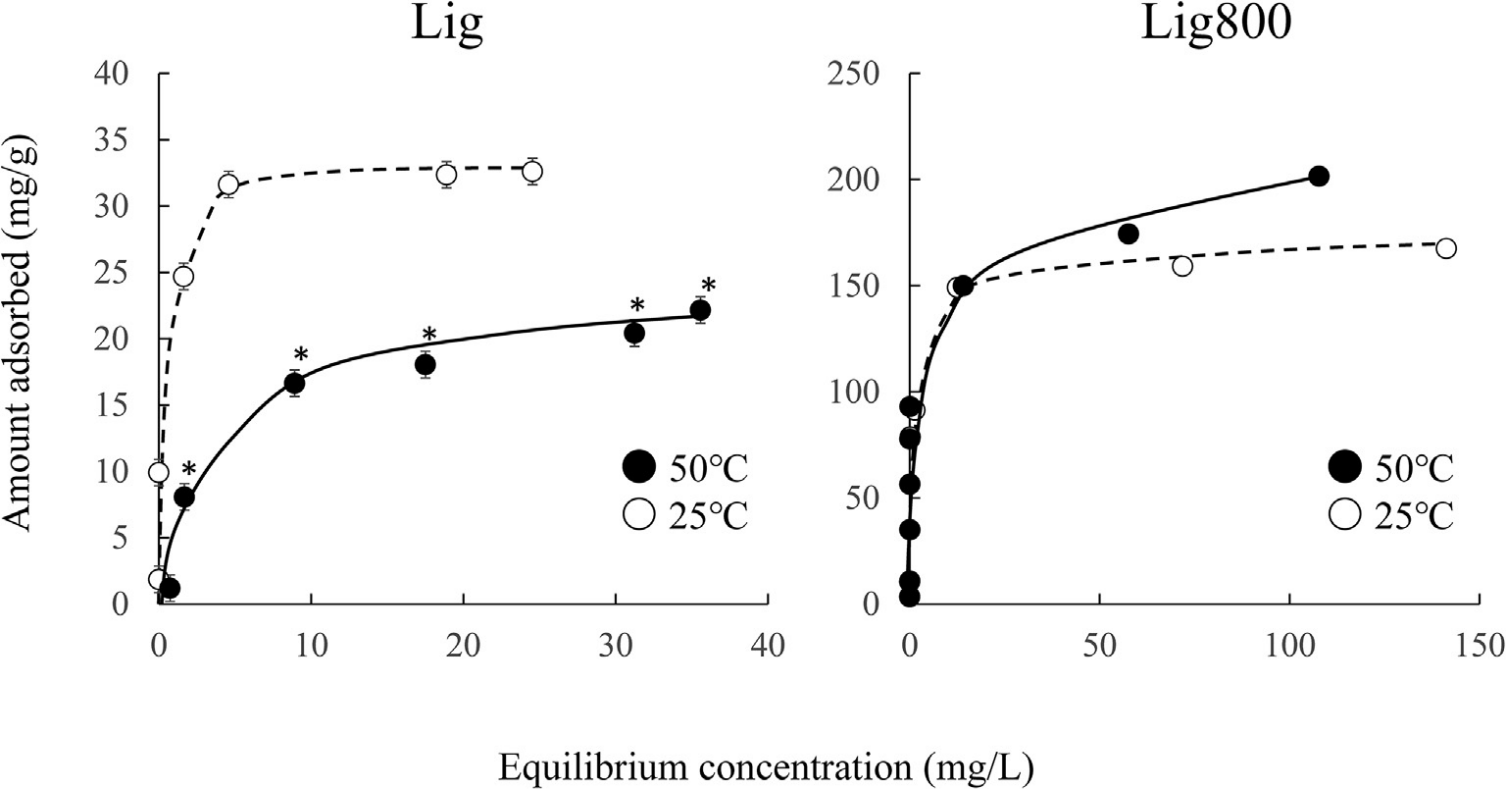
Adsorption isotherms of Cd(II) using Lig and Lig800. Initial concentration: 5–50 and 5–300 mg/L, solvent volume: 50 mL, adsorbent: 0.05 g, pH: 5.3–5.6, contact time: 24 h, temperature: 25 °C, agitation speed: 100 rpm, ∗*p* < 0.05 *vs.* 25 °C, ● 50 °C, ○ 25 °C.

**Figure 5:**
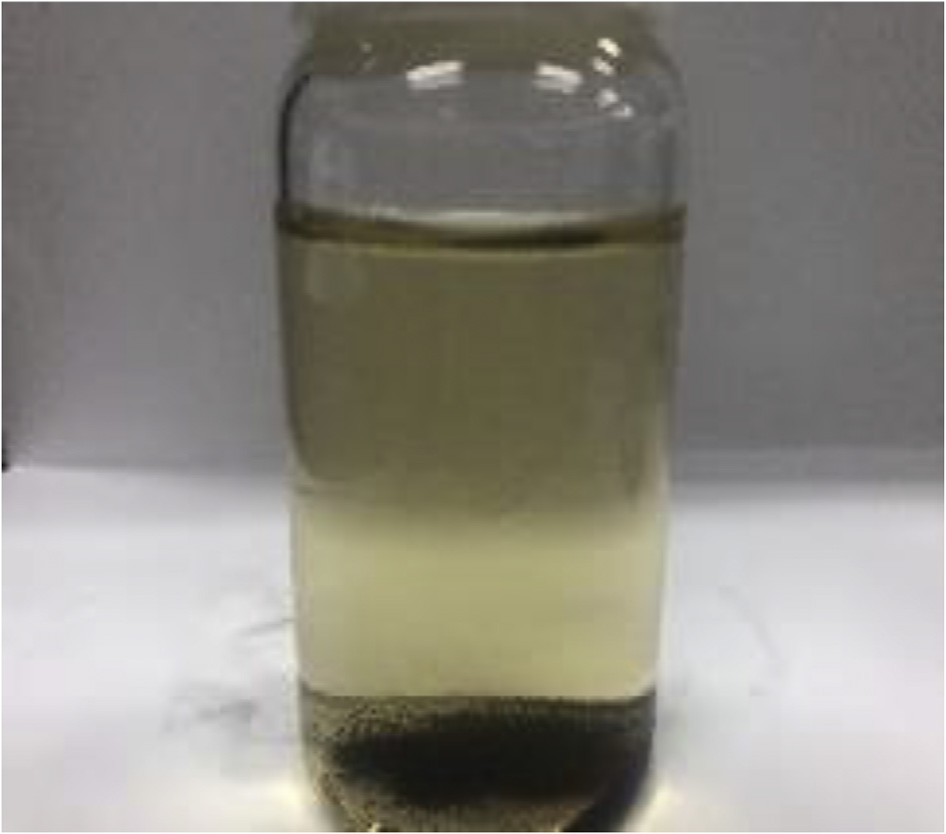
Photograph of sample solution after adsorption of Cd(II) using Lig. Initial concentration: 100 mg/L, solvent volume: 50 mL, adsorbent: 0.05 g, contact time: 24 h, temperature: 25 °C, agitation speed: 100 rpm.

Figure 4 exhibits the adsorption isotherms of Cd(II) using Lig and Lig800. The amount of Cd(II) adsorbed using Lig or Lig800 either decreased or increased by increasing the adsorption temperature (the adsorbed quantity of Cd(II) was less for Lig than for Lig800). These results suggest that the adsorption mechanism of Cd(II) by Lig was different Lig800. From Figure 2, the surface functional groups of Lig are evident; conversely, those of Lig800 were not present in this study. Therefore, the surface functional groups of Lig and the pore volume of Lig800 were strongly related to the adsorption of Cd(II). Various studies previously reported that the adsorption of metal ions (e.g., Cd(II)) on virgin lignin was related to oxygen-containing functional groups (Guo et al., 2008; Mohan et al., 2006; Supanchaiyamat et al., 2019); similar trends were observed in this study. Additionally, light brown dyes were released from Lig after the adsorption of Cd(II) (Figure 5); the phenol group dyes were determined by the 4-aminoantipyrine method (Morita and Nakamura, 2010). In this study, oxygen-containing functional groups, such as phenol groups, affected the adsorption capability of Cd(II) using Lig. Then, light brown dyes (phenol groups) from Lig were released with the increase of adsorption temperature. Therefore, the amount adsorbed at 50 °C was lower than that at 25 °C.

Furthermore, the adsorption isotherm data were fitted to the Langmuir (Eq. (1)) and Freundlich (Eq. (2)) equations. The Langmuir equation is based on the assumption of a homogeneous adsorbent. Conversely, the Freundlich equation is an empirical equation (homogeneous adsorbent).

(1) $$\frac{1}{q}=\frac{1}{q_{max}}+\left(\frac{1}{K_{L}q_{max}}\right)\left(\frac{1}{C}\right)$$

(2) $$\text{log}q=\frac{1}{n}\text{log}C+\text{log}K_{F}$$

where *q* is the quantity of Cd(II) that was adsorbed (mg/g), *q*_max_ is the maximum quantity of Cd(II) adsorbed (mg/g), *K*_*L*_ is the Langmuir isotherm constant (L/mg), and *C* is the equilibrium concentration (mg/L). In addition, *K*_*F*_ and 1/*n* are the Freundlich isotherm constants (Freundlich, 1906; Langmuir, 1916).

**Figure 6:**
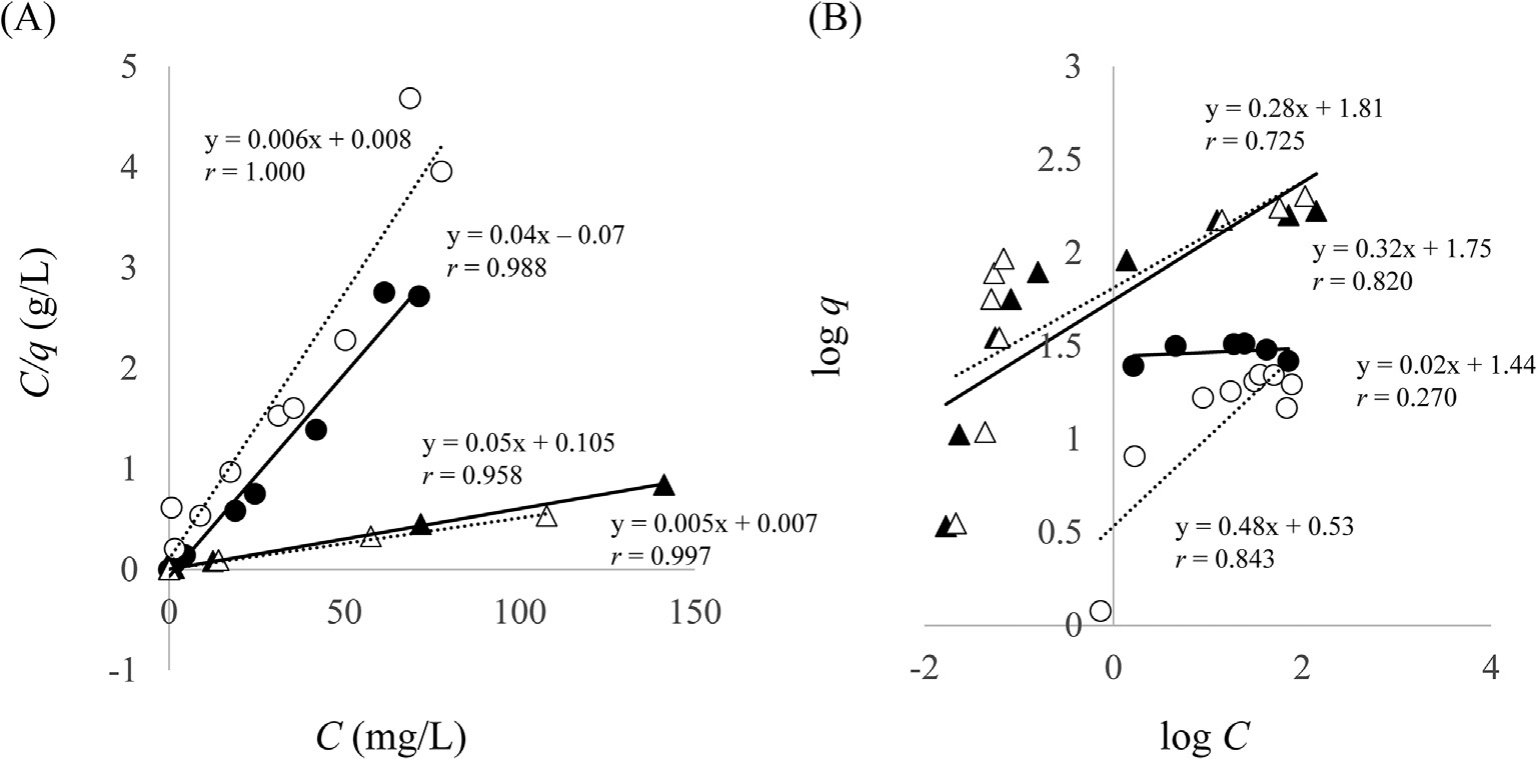
Langmuir isotherm plots (A) and Freundlich isotherm plots (B) for the adsorption of Cd(II). ●: Lig (25 °C, A: y = 0.04x – 0.07, *r* = 0.988, B: y = 0.02x + 1.44, *r* = 0.270), ○: Lig (50 °C, A: y = 0.006x + 0.008, *r* = 1.000, B: y = 0.48x + 0.53, *r* = 0.843), ▲: Lig800 (25 °C, A: y = 0.05x + 0.105, *r* = 0.958, B: y = 0.32x + 1.75, *r* = 0.820), ▵: Lig800 (50 °C, A: y = 0.005x + 0.007, *r* = 0.997, B: y = 0.28x + 1.81, *r* = 0.725).

The correlation coefficients of the Langmuir equation in the values of 0.958–0.988 and 0.997–1.000 for Lig and Lig800, which were higher than those of the Freundlich equation (0.270–0.843 and 0.725–0.820 for Lig and Lig800, respectively) are shown in Table 3 and Figure 6. Therefore, the adsorption isotherm data complied with the monolayer adsorption onto the adsorbent surface with a finite number of identical sites. The trends of *q*_*max*_ in the Langmuir constant using Lig and Lig800 at different temperatures agreed with the adsorption isotherms in Figure 4. Moreover, when the value of 1/*n* in the Freundlich constant is 0.1–0.5, adsorption of Cd(II) occurs easily; when the value of 1/*n* is over 2, adsorption becomes more difficult (Abe et al., 1976). In this study, the value of 1/*n* (0.02–0.48) indicated that Cd(II) was easily adsorbed onto the Lig or Lig800 under our experimental conditions.

**Table 3:** Langmuir and Freundlich constants for the adsorption of Cd(II) using Lig or Lig800.

Samples	Temp. (°C)	Langmuir constants			Freundlich constants		
		*q*_*max*_ (mg/g)	*K*_*L*_ (L/mg)	*r*	log *K*_*F*_	1/*n*	*r*
Lig	25	24.8	0.58	0.988	1.44	0.02	0.270
	50	18.9	0.50	0.958	0.53	0.48	0.843
Lig800	25	166.7	1.25	1.000	1.75	0.32	0.820
	50	196.1	0.75	0.997	1.81	0.28	0.725

Furthermore, isotherm data were analyzed using Temkin and Dubinin–Radushkevich models herein.

The Temkin isotherm model contains a factor which explicitly takes into account the adsorbent–adsorbate interactions. This model assumes that the heat of adsorption in the layer would decrease logarithmically with coverage (Aharoni and Ungarish, 1976; Dada et al., 2012; Temkin and Pyzhev, 1940). The Temkin isotherm model is in the following equations.

(3) $$q_{e}=\;\frac{RT}{b}ln\left(A_{t}C_{e}\right)$$

(4) $$q_{e}=\;\frac{RT}{b_{t}}lnA_{t\;}+\left(\frac{RT}{b}\right)lnC_{e}$$

(5) $$B=\;\frac{RT}{b_{t}}\;$$

(6) $$q_{e}=\;B\;lnA_{t\;}+\left(\frac{RT}{b}\right)lnC_{e}$$

where *A*_*t*_ is the equilibrium binding constant (L/mg), *b*_*t*_ is the adsorption constant, *R* is the universal gas constant (8.314 J/mol K), *T* is the absolute temperature (K), and *B* is the constant related to the heat of adsorption (J/mol).

The Dubinin–Radushkevich model is generally applied to express the adsorption mechanism with Gaussian energy distribution onto a heterogeneous surface (Dada et al., 2012; Dubinin, 1960; Dubilin and Radushkevich, 1947; Gunay et al., 2007).

(7) $$\text{ln}\;q_{e}=\text{ln}\;q_{s}-K_{\mathrm{ad}}\;\varepsilon^{2}$$

(8) $$\varepsilon =RTln\left(1+\;\frac{1}{C_{e}}\right)$$

(9) $$E\;=\;\frac{1}{\sqrt{2K_{ad}}}$$

where *q*_*e*_ is the quantity adsorbed (mg/g), *q*_*s*_ is the theoretical saturated quantity adsorbed (mg/g), and *K*_*ad*_ and *ϵ* are the Dubinin–Radushkevich isotherm constants (mol^2^/kJ^2^), respectively.

**Figure 7:**
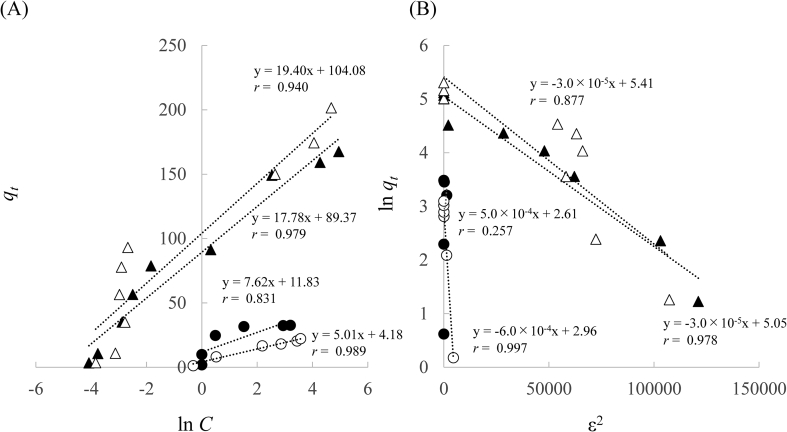
Temkin isotherm plots (A) and Dubinin–Radushkevich isotherm plots (B) for the adsorption of Cd(II). ●: Lig (25 °C, A: y = 7.62x + 11.83, *r* = 0.831, B: y = 5.0×10^−4^x + 2.61, *r* = 0.257), ○: Lig (50 °C, A: y = 5.01x + 4.18, *r* = 0.989, B: y = -6.0×10^−4^x + 2.96, *r* = 0.997), ▲: Lig800 (25 °C, A: y = 17.78x + 89.37, *r* = 0.979, B: y = -3.0×10^−5^x + 5.05, *r* = 0.978), ▵: Lig800 (50 °C, A: y = 19.40x + 104.08, *r* = 0.940, B: y = -3.0×10^−5^x + 5.41, *r* = 0.877).

The constants of Temkin and Dubinin–Radushkevich models are summarized in Table 4 and Figure 7. Low values of *B* in the Temkin model indicate a weak interaction between Cd(II) and each adsorbent. Therefore, herein, the adsorption capacity of Lig800 is greater than that of Lig. Additionally, the correlation coefficient (0.813–0.989) and value of *B* (5.0–19.4 J/mol) in Lig and Lig800 suggest that the adsorption mechanism was related to the physical adsorption process under our experimental conditions (Nechifor et al., 2015). Additionally, the correlation coefficient in the Dubinin–Radushkevich model was 0.877–0.997 (except for Lig, 25 °C). The value of *q*_*s*_ in Lig800 (19.3 and 224.3 mg/g) was higher than that in Lig (13.6 and 156.3 mg/g), which was in agreement with the adsorption isotherm data in Figure 4. Previous studies have reported that the quantity *K*_*ad*_ can be related to the mean adsorption energy, and then *E* is the free energy for the transfer of 1 mol of metal ion from the infinity of the surface of the adsorbent (Naiya et al., 2009). The value of *E* < 8 kJ/mol is an indication of physisorption (Itodo and Itodo, 2010). In this study, the value of *E* using Lig and Lig800 is approximately 1.0 kJ/mol. Therefore, the adsorption process of Cd(II) using Lig and Lig800 was related to the physisorption.

**Table 4:** Temkin and Dubinin-Radushkevich constants for the adsorption of Cd(II) using Lig or Lig800.

Samples	Temp. (°C)	Temkin constants				Dubinin-Radushkevich constants			
		*A*_*t*_ (L/mg)	*b*_*t*_	*B* (J/mol)	*r*	*q*_*s*_ (mg/g)	*K*_*ad*_ (mol^2^/kJ^2^)	*E* (kJ/mol)	*r*
Lig	25	4.73	325.3	7.6	0.831	13.6	-5.0×10-4	1.00	0.257
	50	2.30	494.8	5.0	0.989	19.3	1.2×10-3	0.99	0.997
Lig800	25	152.3	151.0	17.8	0.979	156.3	6.0×10-5	0.99	0.978
	50	213.6	138.4	19.4	0.940	224.3	6.0×10-5	0.99	0.877

**Figure 8:**
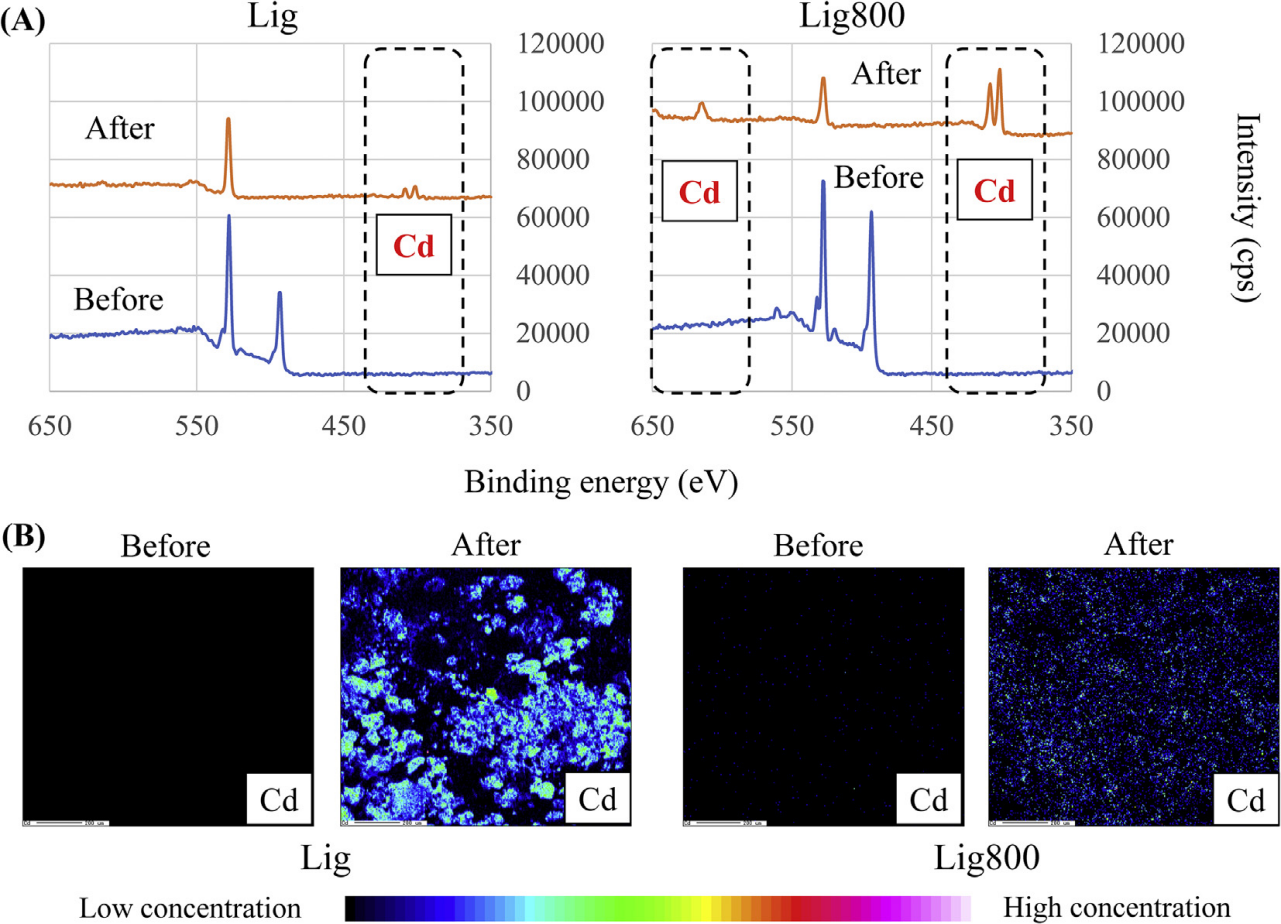
Binding energy (A) and elemental distribution (B) before and after adsorption of Cd(II).

Additionally, the binding energy and the elemental distribution of Cd(II) before and after adsorption were evaluated to elucidate the adsorption mechanism of Cd(II) using Lig or Lig800 (Figure 8). The new peaks of Cd were detected after adsorption using Lig and Lig800, indicating that Cd(II) was adsorbed onto the adsorbent surface. This phenomenon indicated that the adsorption of Cd(II) using Lig and Lig800 was related to the adsorbent surface. Additionally, the elemental distribution of Cd(II) onto the adsorbent surface was measured, and our demonstration of the intensity of Cd increased after adsorption. These obtained results were supported by the abovementioned discussion.

### 3.4 Effect of contact time on the adsorption of Cd(II)

**Figure 9:**
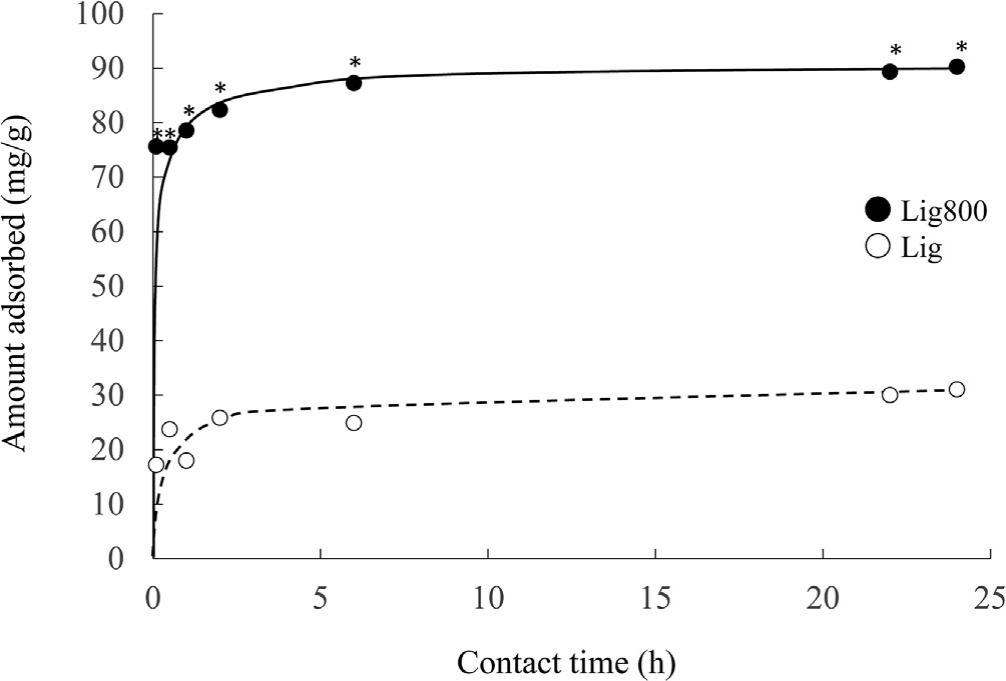
Effect of contact time on the adsorption of Cd(II) using Lig and Lig800. Initial concentration: 100 mg/L, solvent volume: 50 mL, adsorbent: 0.05 g, pH: 5.3, contact time: 1 and 10 min, 0.5, 1, 2, 6, 22, and 24 h, temperature: 25 °C, agitation speed: 100 rpm, ∗*p* < 0.05 *vs.* Lig, ● Lig800, ○ Lig.

The effects of the contact time on the adsorption of Cd(II) using Lig and Lig800 are shown in Figure 9. The adsorption equilibrium of Cd(II) using Lig and Lig800 was reached within approximately 6 h (Lig < Lig800). To assess the efficiency of Lig and Lig800 for the Cd(II) removal from the aqueous phase, the adsorption kinetics were evaluated using a pseudo-first-order model (Eq. (10)) or pseudo-second-order model (Eq. (11)) (Ho and McKay, 1999; Lagergren, 1898).

(10) ln(*q*_*e,exp*_−*q*_*t*_) = ln*q*_*e,cal*_−*k*_1_*t*

(11) $$\frac{t}{q_{t}}=\frac{t}{q_{e,cal}^{2}}+\frac{1}{k_{2}\times q_{e,cal}^{2}}$$

where *q*_*e,exp*_ and *q*_*t*_ are the quantities of Cd(II) adsorbed at equilibrium and at time *t* (mg/g), respectively; *k*_1_ (1/h) and *k*_2_ (mg/μg/h) are rate constants of the pseudo-first-order and pseudo-second-order models, respectively.

**Figure 10:**
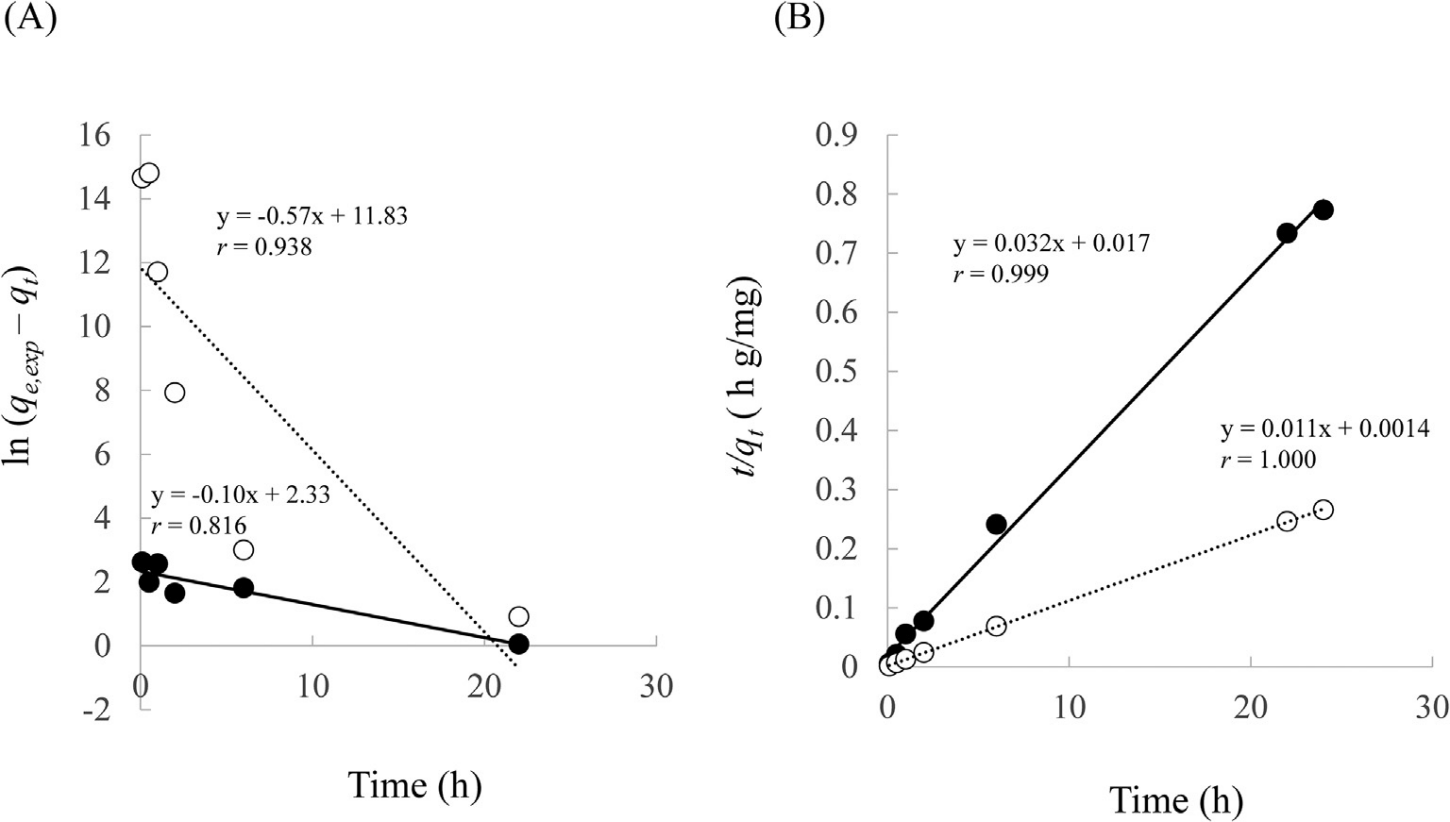
Pseudo-first-order plots (A) and Pseudo-second-order plots (B) for the adsorption of Cd(II). ●: Lig (A: y = -0.10x + 2.33, *r* = 0.816, B: y = 0.032x + 0.017, *r* = 0.999), ○: Lig800 (A: y = -0.57x + 11.83, *r* = 0.938, B: y = 0.011x + 0.0014, *r* = 1.000).

The kinetic constants for the Cd(II) adsorption using Lig and Lig800 are shown in Table 5 and Figure 10. The correlation coefficients of the pseudo-second-order model are in the values of 0.999 and 1.000 for Lig and Lig800, respectively, which were greater than those of the pseudo-first-order model (0.938 and 0.816 for Lig and Lig800, respectively). Moreover, the values of *q*_*e,exp*_ (31.0 and 90.3 mg/g for Lig and Lig800, respectively) were similar to those of *q*_*e,cal*_ (31.06 and 90.09 mg/g for Lig and Lig800, respectively) in the pseudo-second-order equation. These results indicate that adsorption kinetics data were more fitted to the pseudo-second-order model than the pseudo-first-order model. Therefore, the Cd(II) adsorption using Lig and Lig800 was controlled by chemical adsorption under our experimental condition.

**Table 5:** Pseudo-first-order model and pseudo-second-order model constants for the adsorption of Cd(Ⅱ) using Lig and Lig800.

Samples	*q*_*e,exp*_ (mg/g)	Pseudo-first-order model			Pseudo-second-order model		
		*k*_1_ (hr^-1^)	*q*_*e,cal*_ (mg/g)	*r*	*k*_2_ (g/mg/hr)	*q*_*e,cal*_ (mg/g)	*r*
Lig	31.0	0.10	0.10	0.938	0.06	31.06	0.999
Lig800	90.3	0.57	0.00	0.816	0.09	90.09	1.000

Previous studies reported that the value of *k*_2_ in the pseudo-second-order model depended on the experimental conditions such as initial concentration, solvent volume, pH in a solution, temperature, and agitation speed (Sen Gupta and Bhattacharyya, 2011; Zhang et al., 2016). A lower *k*_2_ value implies that a longer time is required to reach equilibrium (Sen Gupta and Bhattacharyya, 2011; Soetaredio et al., 2013). In this study, the values of *k*_2_ using Lig and Lig800 were 0.06 and 0.09 g/mg/h, respectively, indicating that the time to reach the adsorption equilibrium did not differ between them under our experimental conditions.

Then, the kinetics data were analyzed using Elovich and Boyd models. The Elovich model predicts the mass and surface diffusion and the activation and deactivation energy of a system. One of the most useful models assumes the rate of adsorption of solute decreases exponentially as the amount of adsorbed solution increases (Juang and Chen, 1997; Kajjumba et al., 2018; Wu et al., 2009). The Elovich kinetic model, in its nonlinear and linear form, is expressed as follows (Aharoni and Ungarish, 1976).

(12) *q*_*t*_ = β ln (αβ*t*)

(13) *q*_*t*_ = 1/β ln (αβ) + 1/β ln *t*

where *q*_*t*_ is the quantity adsorbed at time *t* (mg/g), α is the constant related to chemisorption rate, and β is a constant which depicts the extent of surface coverage. These constants were determined by the graph of *q*_*t*_ *vs.* ln *t*.

Subsequently, Boyd developed the rate-controlling step in film diffusion. This model indicates that the boundary layer surrounding the adsorbent has a greater effect on the diffusion of solute (Boyd et al., 1947).

(14) $$F\;=\;\frac{q_{t}}{q_{e}}=\;1-\;\frac{6}{\pi^{2}}\;\sum_{1}^{\infty }\left(\frac{1}{n^{2}}\right)exp\left(-n^{2}B\right)$$

*B* can be calculated using the integrated Fourier transform of the following equations.

(15) $$\text{0}\leq \text{F}\leq 0.85\text{: }B=2\pi -\;\frac{F\pi^{2}}{3}-2\pi \;{\left(1-\;\frac{F\pi }{3}\right)}^{1/2}$$

(16) 0.86 ≤ *F* ≤ 1: *B* = -0.4977 - ln (1–*F*)

where *B* is related to the effective diffusion coefficient (*D*_*i*_) and particle radius as *B* = π^2^*D*_*i*_/*R*^2^ (Rui et al., 2014; Tsibranska and Hristova, 2011).

**Figure 11:**
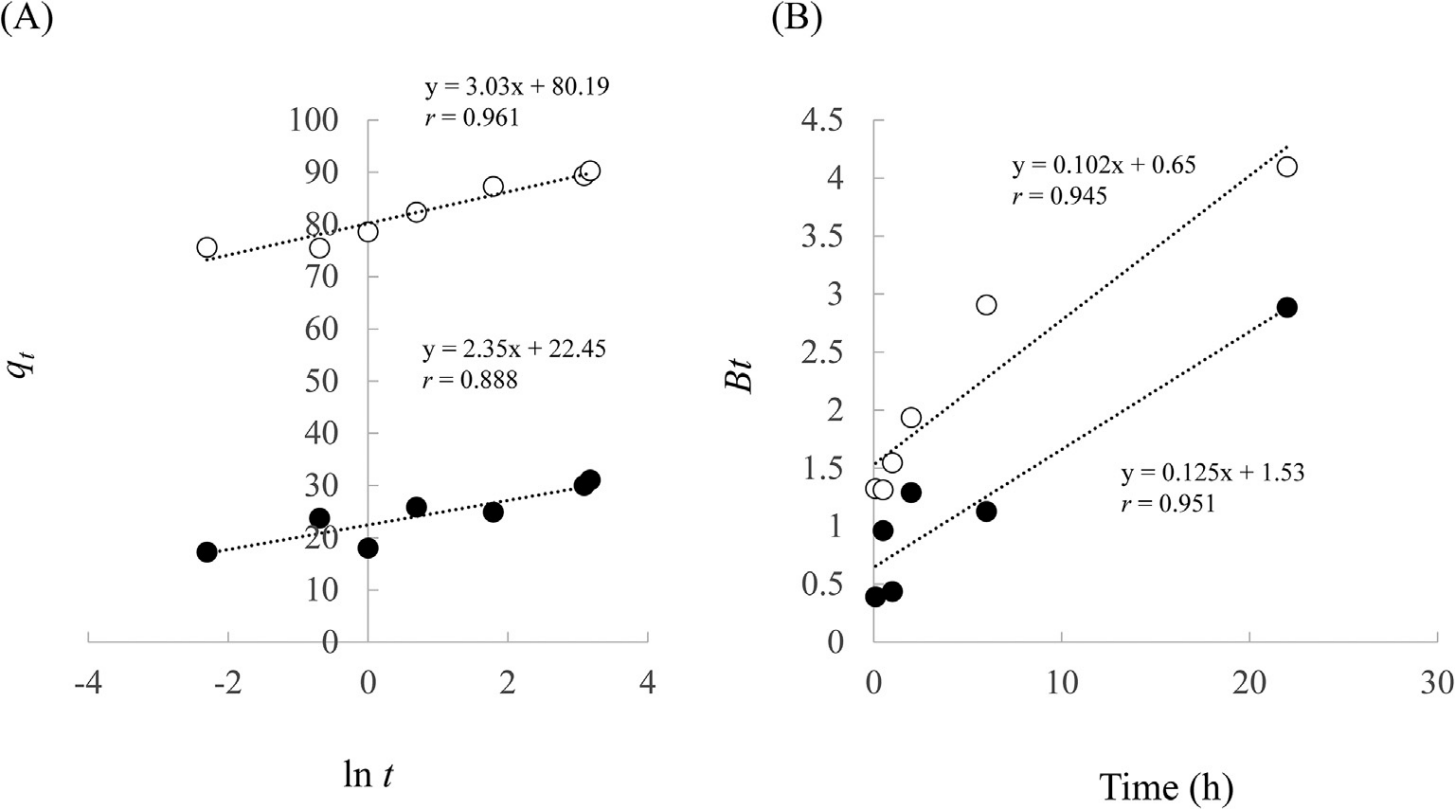
Elovic model plots (A) and Boyd model plots (B) for the adsorption of Cd(II). ●: Lig (A: y = 2.35x + 22.45, *r* = 0.888, B: y = 0.125x + 1.53, *r* = 0.951), ○: Lig800 (A: y = 3.03x + 80.19, *r* = 0.961, B: y = 0.102x + 0.65, *r* = 0.945).

The constants in Elovich and Boyd model are shown in Table 6 and Figure 11. The Elovich model is suitable to describe the adsorption process that is concerned with the nature of chemical adsorption (Juang and Chen, 1997; Wu et al., 2009). The correlation coefficient in Elovich model was 0.888 and 0.961 for Lig and Lig800, respectively. The constant α is related to the chemisorption rate. It can be seen that the higher α showed high adsorption capacity of Cd(II) from the aqueous solution. These results suggest that more than one mechanism governed the adsorption of Cd(II) onto Lig and Lig800 in this study (Ahmad et al., 2015). Next, the correlation coefficients in Lig and Lig800 were 0.945 and 0.951, respectively. Moreover, the Boyd plots suggested that the rate-determining step is the external mass transfer since the plots were linear and do not pass through origin under our experiment (Nethaji et al., 2013). The Boyd model usually describes the rate-limiting step as intraparticle diffusion; otherwise file diffusion model governs the process. However, when particles reached the surface of the adsorbent, film diffusion is the limiting step during the initial stages of the adsorption process (Kajjumba et al., 2018).

**Table 6:** Elovich model and Boyd model constants for the adsorption of Cd(Ⅱ) using Lig and Lig800.

Samples	Elovic model			Boyd model		
	*α* (mg/g h)	*β* (g/mg)	*r*	*B*	*D*_*i*_*/R*^*2*^	*r*
Lig	3.3×104	0.43	0.888	0.65	0.07	0.945
Lig800	9.4×1011	0.33	0.961	1.53	0.16	0.951

### 3.5 Effect of solution pH on the adsorption of Cd(II)

**Figure 12:**
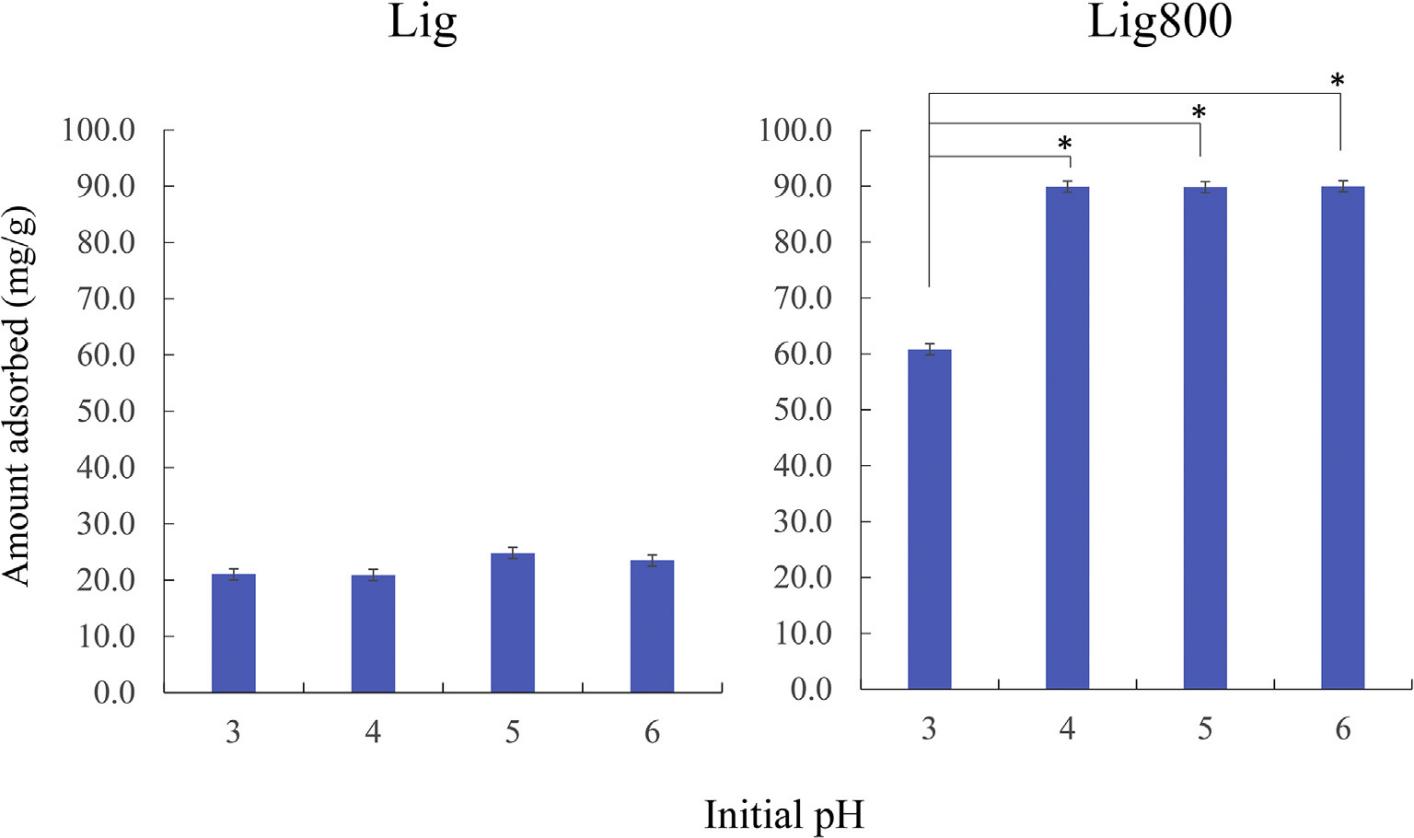
Effect of pH on the adsorption of Cd(II) using Lig and Lig800. Initial concentration: 100 mg/L, solvent volume: 50 mL, adsorbent: 0.05 g, contact time: 24 h, temperature: 25 °C, agitation speed: 100 rpm, ∗*p* < 0.05.

Figure 12 shows the effect of pH on the adsorption of Cd(II) by Lig and Lig800. Two factors mainly affect the adsorption capability of metal ions from an aqueous solution. One is the solubility or distribution of target metal ions in a solution, whereas the other is the charge of the adsorbent surface in the solution. Additionally, Cd(II) formation was present as Cd(II), Cd(OH)^+^, Cd(OH)_2_, Cd(OH)_3_^-^, and Cd(OH)_4_^2^,^-^ depending on the pH of the solution. In addition, the precipitation of Cd(II), such as Cd(OH)_2_, easily occurred at a pH of above approximately 8.0 (Borah and Sennapati, 2006; Mohan et al., 2006; Nazarenko et al., 1979). Therefore, a solution pH from 3 to 6 was selected in our experiment. When using Lig, the quantities adsorbed at pH values of 3 and 4 slightly decreased than those at pH values of 5 and 6. In Sections 3.2*.* and 3.3., the surface functional groups, especially for oxygen-containing functional groups (carboxyl or hydroxyl groups), were an important factor for the Cd(II) removal from the aqueous phase. Therefore, the repulsion between Cd(II) and proton H^+^ easily occurred in acidic conditions. Conversely, the repulsion interaction was weaker with the increase of solution pH (increasing hydroxyl ions). Similar trends were reported by previous literature (Hasan et al., 2006; Horsfall Jr. and Abia, 2003; Kadirvelu and Namasivayam, 2003; Memon et al., 2007; Naiya et al., 2009).

However, when using Lig800, the amount adsorbed at a pH greater than 4 was clearly greater than that at a pH of 3. A previous study had reported that the point of zero charge of calcined lignin in the aqueous phase is at a pH of approximately 2–3 (Berrima et al., 2016). These results indicate the positive and negative charge of the Lig800 surface at a pH of approximately 3 and over a pH of 3 in a solution, respectively. Moreover, there are a number of protons in this acidic condition. Therefore, the repulsion between Cd(II) and the Lig800 surface occurred strongly in a pH of approximately 3 in our experimental condition. This mechanism was supported by a previous study (Berrima et al., 2016).

### 3.6 Desorption capability of Cd(II) from Lig800 using hydrochloric acid solution

To assess the biosorbent regeneration for use in multiple ad-desorption cycles, fundamental experiments were conducted in this study. Many kinds of desorption solutions were used by previous studies for the desorption of metal ions from biosorbents. Our previous study also reported that hydrochloric acid was one of the most useful desorption solutions (Celik and Demirbas, 2005; Ogata et al., 2014). Additionally, a hydrochloric acid solution at over 10 mmol/L affected the structure of lignin (Njikam and Schiewer, 2012). Therefore, hydrochloric acid solutions at different concentrations (1 and 10 mmol/L) were selected for the desorption of Cd(II) from Lig800 in this experiment. First, the amount of Cd(II) adsorbed was measured as 134.3 mg/g in the adsorption experiment. The desorption percentage of Cd(II) using hydrochloric acid solutions at 1 and 10 mmol/L was 70.5 % and 92.6 %, respectively. Additionally, the optimal pH condition for adsorption of Cd(II) using Lig800 was approximately 4 or above (Figure 12, *3.5. Effect of solution pH on the adsorption of Cd(II)*). Therefore, the acidic condition (solution pH: below 4) was suitable for the desorption of Cd(II) using Lig800 under our experiment. Thus, the desorption percentage using 10 mmol/L hydrochloric acid was higher than that using 1 mmol/L. The desorption mechanism of metal from bio-sorbent using a desorption solution may involve ion exchange or complexation, among other things. Further studies will be necessary to elucidate the desorption mechanism of Cd(II) from lignin using a hydrochloric acid solution.

## 4 Conclusion

The specific surface area and pore volumes of Lig800 were greater than those of other experimental adsorbents. Additionally, the quantity of Cd(II) adsorbed by Lig800 was also higher than that of other experimental adsorbents. The specific surface area and pore volumes were strongly related to the capability for adsorption of Cd(II). The binding energy and distribution of Cd(II) after adsorption were first detected through this study. Finally, it was determined that a hydrochloric acid solution could be useful for desorbing Cd(II) from Lig800. Our study determined that Lig800 was useful for the removal of Cd(II) in an aqueous phase.

## Declarations

### Author contribution statement

Fumihiko Ogata: Conceived and designed the experiments; Analyzed and interpreted the data; Wrote the paper.

Eri Nagahashi, Hirona Miki: Performed the experiments; Analyzed and interpreted the data.

Chalermpong Saenjum: Contributed reagents, materials, analysis tools or data.

Takehiro Nakamura: Performed the experiments.

Naohito Kawasaki: Conceived and designed the experiments; Wrote the paper.

### Funding statement

This research did not receive any specific grant from funding agencies in the public, commercial, or not-for-profit sectors.

### Competing interest statement

The authors declare no conflict of interest.

### Additional information

No additional information is available for this paper.
